# Colloidal Synthesis of Gold Semishells

**DOI:** 10.1002/open.201200002

**Published:** 2012-03-13

**Authors:** Denis Rodríguez-Fernández, Jorge Pérez-Juste, Isabel Pastoriza-Santos, Luis M Liz-Marzán

**Affiliations:** [a] Departamento de Química Física, Universidade de Vigo36310 Vigo (Spain) E-mail: lmarzan@uvigo.es

**Keywords:** asymmetric syntheses, colloids, Janus particles, semishells

## Abstract

This work describes a novel and scalable colloid chemistry strategy to fabricate gold semishells based on the selective growth of gold on Janus silica particles (500 nm in diameter) partly functionalized with amino groups. The modulation of the geometry of the Janus silica particles allows us to tune the final morphology of the gold semishells. This method also provides a route to fabricating hollow gold semishells through etching of the silica cores with hydrofluoric acid. The optical properties were characterized by visible near-infrared (vis-NIR) spectroscopy and compared with simulations performed using the boundary element method (BEM). These revealed that the main optical features are located beyond the NIR region because of the large core size.

## Introduction

The recent advances in the preparation of colloidal particles comprising different properties (such as chemistry, polarity, and optical and magnetic properties) at opposite sides, commonly known as Janus particles,[1] have opened new avenues toward the development of advanced materials. The reduced symmetry in these materials provides them with fascinating properties and, therefore, with potential applications in various fields including electronic paper,[2] photonic materials,[3] imaging probes,[4] and sensors.[3] Exciting future applications are expected in connection with their interesting self-assembly behavior, since the presence of distinct areas within Janus particles renders them powerful building blocks for the fabrication of novel structures.[5, 6, 7]

Janus particles based on a dielectric/polymeric core partially covered with a metal are commonly referred to as semishells, but different terms have also been used, such as half-shells or nanocups, depending on the metal surface coverage. More recently, the expression “plasmonic patchy particles with reduced symmetry” has been used for particles with a more heterogeneous metal distribution on the core.[8] It has been demonstrated that plasmonic semishells present unusual and unique optical properties due to their reduced symmetry.[9] The inherent asymmetry of these systems leads to the presence of two types of dipole plasmon modes: a blue-shifted axial mode and a red-shifted transverse mode. While the blue-shifted axial mode is purely electric, calculations predict that there is also a significant magnetic component in the red-shifted transverse mode.[10, 11, 12] These dipolar resonances display different light-scattering characteristics that are strongly influenced by the angle and polarization of the incident light and by variations in the dielectric environment. Additionally, the optical response can be easily tailored from the visible through to the near-infrared (NIR) by tuning the core radius, metal thickness, and surface coverage. Altogether, plasmonic semishells offer a variety of new possibilities for the fabrication of metamaterials with negative refractive indices at NIR and visible frequencies,[13] or of surface-enhanced Raman scattering (SERS) probes.[14]

On the basis of the above-described properties, there is a need to develop novel and efficient synthetic methods that allow us to control the morphological and compositional parameters of semishells. Two fundamentally different approaches have been applied so far to fabricate plasmonic nanostructures with reduced symmetry: the deposition of a metal onto a close-packed colloid monolayer, using electroless plating, e-beam evaporation or sputtering; and the anisotropic etching, via ion milling, Ar-ion plasma or electron-beam-induced ablation of full nanoshells pre-deposited on a substrate.[9] However, the application of colloidal synthesis to the fabrication of plasmonic semishells has been far less explored. The closest example is probably the recent work by Klupp Taylor et al., who have reported the fabrication of Ag patchy particles with tunable optical properties via electroless deposition onto nonfunctionalized colloidal silica spheres.[8, 15] Although this method does show a certain degree of control over metal coverage, it has not demonstrated the formation of solid semishells. Here, we present an alternative method to the design and fabrication of gold semishells using a colloid chemistry route based on the use of pre-formed Janus silica particles as templates, where the selective growth of Au can only take place on the functionalized areas. A similar approach was employed by Perro et al. for the production of large quantities of Janus particles, but they did not complete the process for the synthesis of Au semishells.[16] The growth of Au semishells is carried out by employing the method developed by Halas et al.[17] for Au-nanoshell synthesis, however, partial coverage is achieved by selective partial functionalization of the silica particles with amino groups on one side. Such Janus silica particles were synthesized using a masking process based on Pickering emulsions, recently reported by Granick et al.[18] The final morphology of the Au semishells can thus be tuned through the geometry of the Janus silica template. Additionally, we demonstrate the fabrication of hollow Au semishells by simply etching the silica cores with a hydrofluoric acid solution. The optical response of the resulting semishells was characterized by visible near-infrared (vis-NIR) spectroscopy and compared to calculations of the extinction cross section using the boundary element method (BEM).[19, 20] This allowed us to analyze the effect of Au surface coverage, shell thickness and core diameter.

## Results and Discussion

**Scheme 1:**
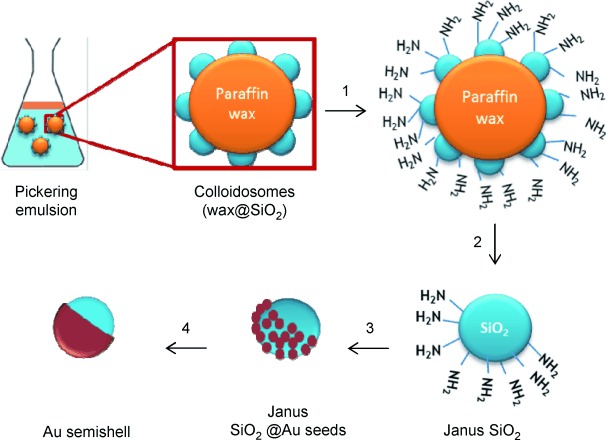
Schematic representation of the synthetic route to form gold semishells: Gas phase amine functionalization of silica particles attached to wax colloidosomes (1), dissolution of the wax and release of the Janus silica particles (2), adsorption of gold seeds through amine groups (3), formation of Au semishells through seeded growth (4).

A schematic representation of the general strategy used for the fabrication of Au semishells is shown in Scheme 1. The process involves two main steps: the preparation of Janus silica particles partially functionalized with amine groups and the seeded growth of the Au semishells. Initially, Janus particles were synthesized following the toposelective modification method.[18, 21] More specifically, 495 nm Stöber silica spheres were adsorbed onto the liquid–liquid interface of an emulsion formed by molten paraffin wax and water.[21] Once the emulsion cooled, the silica particles were locked at the interface, forming stable colloidosomes (see scanning electron microscope (SEM) images in Figure S1, Supporting Information), so that the silica surface embedded into the wax was protected from further chemical modification. The following step was the silanization of the available silica surface (step 1, Scheme 1) using the more volatile (3-ethoxydimethylsilyl)propylamine (APDMES), rather than other common aminosilanes, such as (3-aminopropyl)triethoxysilane (APS), in order to achieve self-assembled monolayers of higher quality.[22] The silane grafting was carried out using a solvent-free method at room temperature[18] comprising the volatilization of APDMES with bubbling N_2_ and the reaction of the produced silane vapor with the dried colloidosomes deposited on a filter funnel (see experimental setup in Figure S2, Supporting Information). This route was preferred over solution-phase silanization, since it proved to have no effect on either the adhesion of the silica particles or the stability of the colloidosomes, while additionally improving the efficiency of silane grafting (Figure S1 in the Supporting Information shows SEM images of colloidosomes before and after the different chemical modification routes). After APDMES deposition, the silica particles can be easily released by dissolving the paraffin wax in chloroform (step 2, Scheme 1), resulting in the formation of Janus silica particles with toposelective amine functionality.

**Figure 1:**
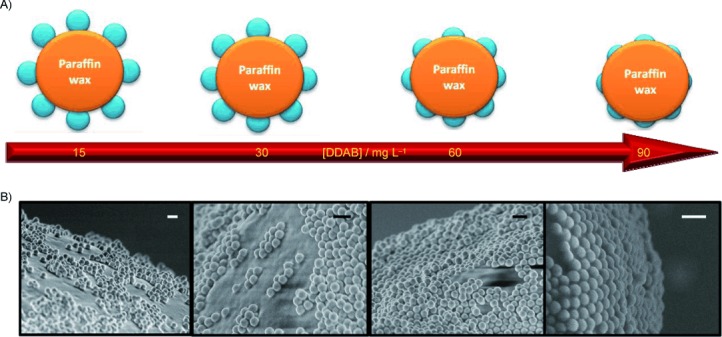
A) Schematic representation of the effect of DDAB concentration on the immersion of silica particles into the wax phase. B) SEM images of the surface of colloidosomes fabricated with approximately 495 nm SiO_2_ particles and DDAB concentrations of 15, 30, 60 and 90 mg L^−1^ (left to right). All scale bars correspond to 1 μm.

Interestingly, the surface composition of the Janus silica particles can be tuned by adding small amounts of didodecyldimethylammonium bromide (DDAB) during the preparation of the Pickering emulsion (Figure 1).[21] The cationic surfactant adsorbs strongly onto the negatively charged surface of the silica particles, changing the effective hydrophobicity of the surface and producing a deeper immersion of the silica into the wax and, therefore, increasing the three-phase contact angle of the particles at the emulsion interface. The SEM micrographs in Figure 1 B show selected areas on wax–silica colloidosomes synthesized in the presence of different amounts of DDAB (15–90 mg L^−1^). It can be seen that silica particles are more deeply embedded into the wax phase at increasing DDAB concentrations. An accurate characterization would require the measurement of the contact angle of the particles at the water–wax interface. This can be indirectly performed by measuring the size of the voids that are left on the wax when the silica particles are detached during the washing step.[21] Experimentally, this measurement is not trivial, since at room temperature the samples might be affected by the electron beam, and therefore, the use of cryo-SEM is required. In this case, the process was even more complicated due to the small dimensions (<500 nm) of the silica particles, resulting in the formation of rather small voids. For a representative sample obtained with a DDAB concentration of 60 mg L^−1^, the average contact angle measured was approximately 68°, which is in agreement with the values reported by Granick et al.[21] (cryo-SEM image is shown in Figure S3, Supporting Information).

**Figure 2:**
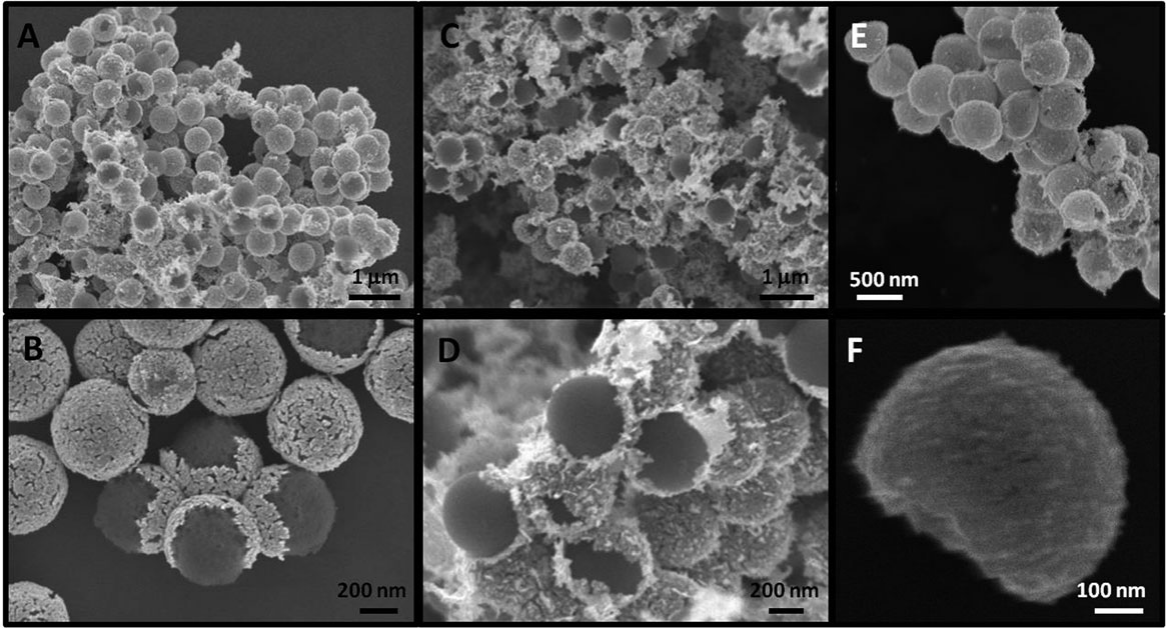
SEM images of Au semishells obtained after six growth steps using Janus silica particles prepared with DDAB concentrations of 60 mg L^−1^ (A and B) and 90 mg L^−1^ (C and D). SEM images of hollow Au semishells upon etching of the silica cores (E and F).

The preparation of Au semishells templated by Janus silica particles with toposelective amine functionality is represented by steps 3 and 4 in Scheme 1. This approach is based on the well-known method for the growth of Au nanoshells developed by Halas et al.[17, 23] Initially, Gold nanoparticles smaller than 3 nm in size were adsorbed onto the functionalized side of the Janus silica particles through a weak covalent bond with the amino groups of the aminosilane.[24] Subsequently, a continuous Au semishell was grown on the gold seeds in a multistep process through the further catalytic reduction of hydrogen tetrachloroaurate (HAuCl_4_) by formaldehyde (see Figure 2). It was found that a sequential growth of the Au semishells was required to obtain uniform and smooth Au surfaces. Another important parameter that had to be taken into account for the fabrication of homogeneous Au semishells was the ratio between Au^3+^ ions and Janus silica particles in each growth step.

While ratios below 10^8^:1 led to uniform Au semishells (Figure 2), a greater excess of Au^3+^ ions resulted in the metal surface of the semishell being uneven and spiked, and gold debris and bare silica particles were also observed, probably due to the rapid reduction of the gold salt (see Figure S4, Supporting Information). These Au semishells sediment after a few minutes, but could be redispersed by applying weak sonication. The random orientation of the Au semishells on the substrate makes the morphological characterization rather difficult. In order to demonstrate the anisotropy of these plasmonic nanostructures, the silica core was dissolved using hydrofluoric acid (see Experimental Section). As clearly shown in Figure 2 E and F, the removal of the dielectric cores led to the formation of hollow metallic semishells. Apart from the possibility of synthesizing hollow nanostructures, another advantage of this colloidal synthetic route is that the Au semishell geometry can be controlled by tuning the concentration of DDAB and, thus, the coverage of amine functionality in the Janus silica particles, as discussed above. This is exemplified in Figure 2, where SEM images are shown of Au semishells fabricated from Janus silica particles obtained with different DDAB concentrations (60 and 90 mg L^−1^). Although the random orientation of the Au nanoshells makes a comparison rather difficult, it is possible to observe that the particles fabricated using 60 mg L^−1^ DDAB (Figure 2 A,B) present a higher metal coverage than those obtained using 90 mg L^−1^ DDAB (Figure 2 C,D).

**Figure 3:**
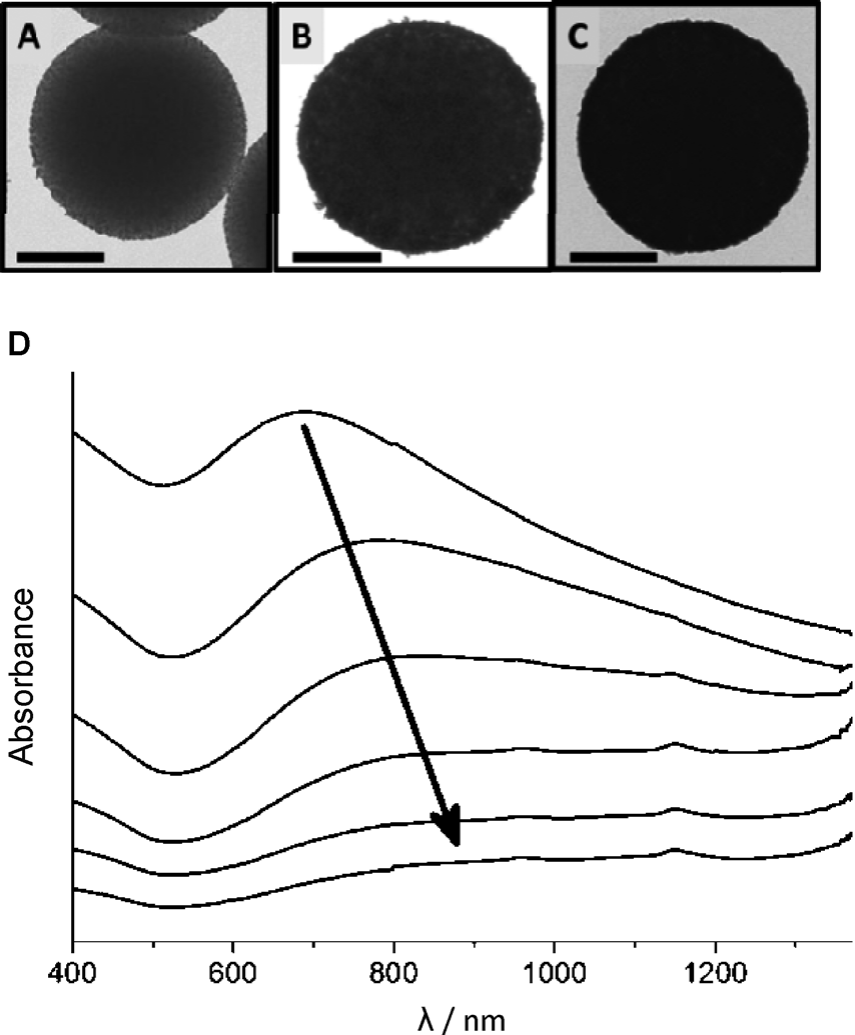
TEM images of Janus silica particles after the A) first, B) third and C) fifth growth step, showing increased Au-surface coverage. All scale bars correspond to 200 nm. D) Evolution of the vis-NIR spectra during a sequential growth (in six steps) of Au seeds attached to 495 nm Janus silica particles to obtain Au semishells. The spectra were shifted to facilitate comparison.

The mechanism of Au-semishell growth is similar to that observed for Au nanoshells by Halas et al.[23] In this multistep process, the small Au seeds initially attached to the Janus silica particles grow larger and ultimately merge to form a continuous and polycrystalline gold semishell (Figure 3 A–C). This process can be followed in vis-NIR absorption spectra measured during Au-semishell growth (in six steps), as shown in Figure 3. Initially, the surface plasmon resonance band shifts to longer wavelengths and broadens due to the growth of the Au seeds and also to the plasmon coupling that arises when the distance between metal particles on the silica surface decreases. Further reduction leads to the formation of a continuous shell and results in a shift of the surface plasmon resonance band beyond the NIR (1400 nm, see discussion below). However, we were not able to resolve these main features experimentally, since the optical characterization is limited to the vis-NIR range (up to about 1400 nm) and only a broad continuum band is observed in this region. The experimental spectra of Au semishells synthesized with 60 or 90 mg L^−1^ of DDAB are similar in that a broad plasmon band in the analyzed region is observed (vis-NIR).

**Figure 4:**
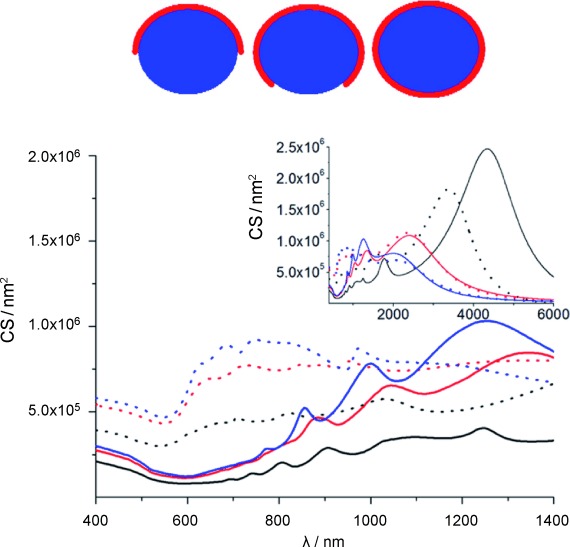
Calculated extinction cross section (CS) spectra for Au semishells with 50 % (—) and 75 % (—) metal coverage, and complete nanoshells (—). The thickness of the gold shells was 10 nm (solid) and 30 nm (dashed). The diameter of the silica cores was 500 nm. The inset shows the same spectra for an extended wavelength range up to 6000 nm. The geometrical models employed in the simulations are shown above.

We attempted to understand the optical properties of the obtained Au semishells through simulations of the optical properties by means of the BEM for numerical resolution of Maxwell's equations.[19, 20] Since the core size and shell thickness are the main parameters affecting the surface plasmon energies of Au nanoshells,[25–27] we modeled different morphologies varying these parameters as well as surface coverage. The geometrical models used for the simulations are shown in Figure 4. The extinction cross sections obtained for Au semishells with silica cores of 500 nm in diameter, metal surface coverage of 50 % and 75 % and Au-shell thicknesses of 10 and 30 nm are plotted in Figure 4. For comparison, the calculated spectra for Au nanoshells (100 % surface coverage) are also included. The most important point to note is that the loss of symmetry (from nanoshell to semishell) produces a strong red-shift of the main plasmon bands, this effect being stronger for lower metal surface coverage. Due to the large dimension of the silica core (500 nm) and the thin Au shell, the main plasmon band in the semishell, arising from transverse dipolar resonances (perpendicular to the axis of symmetry; see Figure S5, Supporting Information), is located beyond the NIR (in the far IR region for 50 % coverage). The optical features observed in the vis-NIR region (broad band) seem to correspond to a less intense axial dipolar mode (resonances parallel to the axis of symmetry and located around 1100 nm; see Figure S5, Supporting Information) and several multipolar plasmon modes. These simulations are in good agreement with the broad bands obtained experimentally (Figure 3) and show that the main plasmon band could be shifted within the NIR (region of interest for biomedical applications) by using smaller Janus silica particles as templates. This was confirmed by calculations for shells and semishells comprising 100-nm silica cores (Figure S6, Supporting Information), which showed that the transverse dipolar modes in that case are located between 750 and 950 nm, depending on the surface coverage and Au-shell thickness.

## Conclusion

We have developed a versatile and easily scalable colloidal strategy for the synthesis of Au semishells based on the use of Janus silica particles as templates to grow uniform metallic shells. Preliminary results show that this method enables the control of the geometry of the plasmonic structures by tuning the Janus silica particle composition through variation of the concentration of the surfactant didodecyldimethylammonium bromide (DDAB), which modulates the penetration of the silica particles at the wax–water interface. Additionally, the silica cores can be readily etched away, leading to the fabrication of hollow Au semishells. The simulation of the optical properties using the boundary element method (BEM) has shown that the main optical features in the case of Au semishells with 500 nm silica cores are located beyond the NIR range and, therefore, cannot be observed experimentally. However, it is possible to fabricate Au semishells with optical properties in the NIR region by simply decreasing the core size.

## Experimental Section

**Chemicals**: HAuCl_4_⋅3H_2_O (99 %) and didodecyldimethylammonium bromide (DDAB, 98 %) were supplied by Sigma. NH_4_OH (28 %), tetraethylorthosilicate (TEOS, 98 %), (3-ethoxydimethylsilyl)propylamine (APMES), paraffin wax (mp=58–62 °C), K_2_CO_3_ (99 %), CHCl_3_, tetrakis(hydroxymethyl)phosphonium chloride (THPC, 80 %), NaOH, NaCl and formaldehyde (H_2_CO, 37 %) were supplied by Aldrich. HF (40 %) was purchased from Panreac (Barcelona, Spain). Abs EtOH (Scharlau, Barcelona, Spain) and Milli-Q™ H_2_O (Millipore, Billerca, MA, USA) were used as solvents. All chemicals were used as received from the supplier.

### Fabrication of Janus silica particles

**Synthesis of SiO_2_** **particles**: The synthesis was performed according to Jiang et al.[17] Briefly, 495 nm silica particles were prepared using a variation of the Stöber method.[29] All glassware was cleaned with an aq HF solution (2 %) before starting. TEOS (0.3 m final concentration) was added to an EtOH solution (250 mL) containing H_2_O (7 M) and NaOH (2 m) under vigorous stirring. When the solution became turbid, stirring was slowed and continued for 8 h. The silica particles were then washed by four cycles of centrifugation (3400 RCF, 20 min) and redispersion in EtOH (60 mL). The nanoparticles were dried in vacuo and stored.

**Preparation of Pickering emulsion**: The emulsions were prepared according to Jiang et al.[17] with some modifications. Freshly prepared aq DDAB (20 mL, 15–90 mg L^−1^) containing silica particles (0.4 g, 495 nm) was heated to 70–75 °C and then added to molten paraffin wax (5 g) at 70–75 °C. The mixture was maintained at 70–75 °C for 1 h until equilibrium was reach, and then cooled to RT. The resulting colloidosomes are visible as solid wax droplets with adsorbed silica particles. To remove unattached and weakly attached silica particles, the colloidosomes were washed multiple times with H_2_O (40 mL) stirring manually for a few seconds, until a colorless solution was observed. Finally, the colloidosomes were filtered and dried in vacuo (30 °C, 300 mbar) for 2 d.

**Surface silanization of colloidosomes**: The surface functionalization was carried out using a gas-phase method reported by Jiang et al.[17] Briefly, a silane vapor, produced by bubbling dry N_2_ (0.25 bar) through APDMES (500 μL), was reacted for 30 min with colloidosomes (∼0.5 g) deposited on a filter funnel (see setup in Figure S2, Supporting Information). The colloidosomes were agitated every 5 min by rolling the filter to obtain a homogeneous functionalization of the surface. Finally, a stream of dry N_2_ was passed through the particles for 30 min to remove excess silane. To release the Janus silica particles from the wax surface, the paraffin was dissolved by washing first with CHCl_3_ (2×30 mL). The particles were then exposed to two cycles of dispersion in EtOH (30 mL) and subsequent centrifugation (2300 RCF, 20 min). Finally, the Janus particles were dried in vacuo and stored.

### Fabrication of Au semishells

Au semishells were prepared by a sequential growth method according to the process reported by Halas et al.[16] with slight modifications as described below.

**Synthesis of sub-3 nm gold nanoparticles**: According to Duff et al.,[29] THPC gold seeds (200 mL, 1–2 nm) were prepared as follows: NaOH (1.2 mL, 1 M) and aq THPC (4 mL, 6.8×10^−2^ m) were added to H_2_O (180 mL) under vigorous stirring. After 5 min, aq HAuCl_4_ (1.53 mL, 0.13 m) was added in one quick motion. The solution changed instantly from colorless to deep brown, was cooled to 4 °C and stored until needed (after 2–8 weeks).

**Preparation of gold plating solution**: An aq solution containing K_2_CO_3_ (1.8 mm) and HAuCl_4_ (44 μM) was prepared and stored at 4 °C for 24 h prior to use. More homogeneous shells were obtained when the plating solution aged for 24–48 h.

**Preparation of the precursor particles**: NaCl (4 mL, 1 m) and a dispersion of Janus silica particles in EtOH (5–10 mg, 300 μL) were added to freshly sonicated (1 min) gold seeds (40 mL). The mixture was sonicated for 2 min and allowed to reach equilibrium for 12–24 h. The particles were centrifuged (850 RCF, 20 min) and washed with H_2_O (3×10 mL). Finally, the precursor particles were dispersed in H_2_O (7×10^9^ particles mL^−1^) and stored at 4 °C. The particles could be used for at least 48 h to obtain complete Au semishells.

**Growth of Au semishells**: All solutions were brought to 25 °C for 30 min before use. First, the plating solution (96.4 mL) was mixed with the aq dispersion of precursor particles (3.6 mL, 7×10^9^ particles mL^−1^), giving a 10^8^:1 ratio of gold ions to precursor particles. Subsequently, H_2_CO (500 μL) was added to the mixture under vigorous stirring. After 15 s, stirring was slowed and the reaction was allowed to proceed for 10–15 min. Before starting the second growth step, the particles were centrifuged (850 RCF, 30 min) and redispersed in H_2_O (3.6 mL). After five growth steps, the resulting Au semishells were washed by four cycles of centrifugation (850 RCF, 30 min) and redispersion in H_2_O (20 mL) to ensure the complete removal of excess K_2_CO_3_ and H_2_CO. The Au semishells were stored in H_2_O at RT.

### Synthesis of hollow metallic semishells

Hollow plasmonic structures were obtained by exposure of Au semishells to aq HF (10 %) for 6 h. The particles were then washed by three cycles of centrifugation (850 RCF, 30 min) and redispersion in H_2_O (5 mL).

### Particle characterization

Visible near-infrared (vis-NIR) spectra were measured in 1 cm path length quartz cuvettes using a Cary 5000 UV-vis-NIR spectrophotometer (Agilent Technologies, Santa Clara, CA, USA). A JEM 1010 transmission electron microscope (TEM, JOEL, Tokyo, Japan) operating at an acceleration voltage of 100 kV was used for particle characterization. Scanning electron microscope (SEM) images were obtained using a JSM-6700F FEG SEM (JOEL, Tokyo, Japan).

### Optical modeling

Simulations of optical spectra were performed using the boundary element method (BEM)[19, 20] for Au semishells and nanoshells with a silica core and H_2_O as solvent. The edge of the semishell was described as an arc to avoid sharp corners that can cause numerical problems. The dielectric data for Au and silica were taken from Johnson and Christy[30] and Palik,[31] respectively. Convergence was achieved with 200 (140) parameterization points for the 500 (100) nm core and 160 (120), 220 (160), 160 (160) points for 50 %, 75 % and 100 % surface coverage, respectively.

### Supporting Information

The following are available as Supporting Information: SEM micrographs showing a comparison between different silanization conditions (Figure S1). Photograph of the gas-phase silanization setup (Figure S2). Cryo-SEM image showing voids left by removal of silica particles from the paraffin wax during washing steps (Figure S3). SEM micrograph showing spikes formed on gold semishells when the growth ratio was too high (Figure S4). BEM simulations of 500 nm gold semishells and complete shells (Figure S5). BEM simulations of 100 nm gold semishells with a 10 nm gold layer showing the axial and transverse modes.

## Supplementary material

Detailed facts of importance to specialist readers are published as ”Supporting Information”. Such documents are peer-reviewed, but not copy-edited or typeset. They are made available as submitted by the authors.
