# Supplementary material for: Colloidal Synthesis of Gold Semishells
Source: ChemistryOpen. 2012 Mar 13;1(2):90–5. doi: 10.1002/open.201200002 (PMC3922459; doi:10.1002/open.201200002)
Supplement: Supplementary file 1 [file open0001-0090-SD1.pdf]

ChemistryOPEN

Supporting Information

© Copyright Wiley-VCH Verlag GmbH & Co. KGaA, 69451 Weinheim, 2012

Colloidal Synthesis of Gold Semishells

Denis Rodríguez-Fernández, Jorge Pérez-Juste, Isabel Pastoriza-Santos, and Luis M. Liz-Marzán*[a]

open_201200002_sm_miscellaneous_information.pdf

Supporting Information

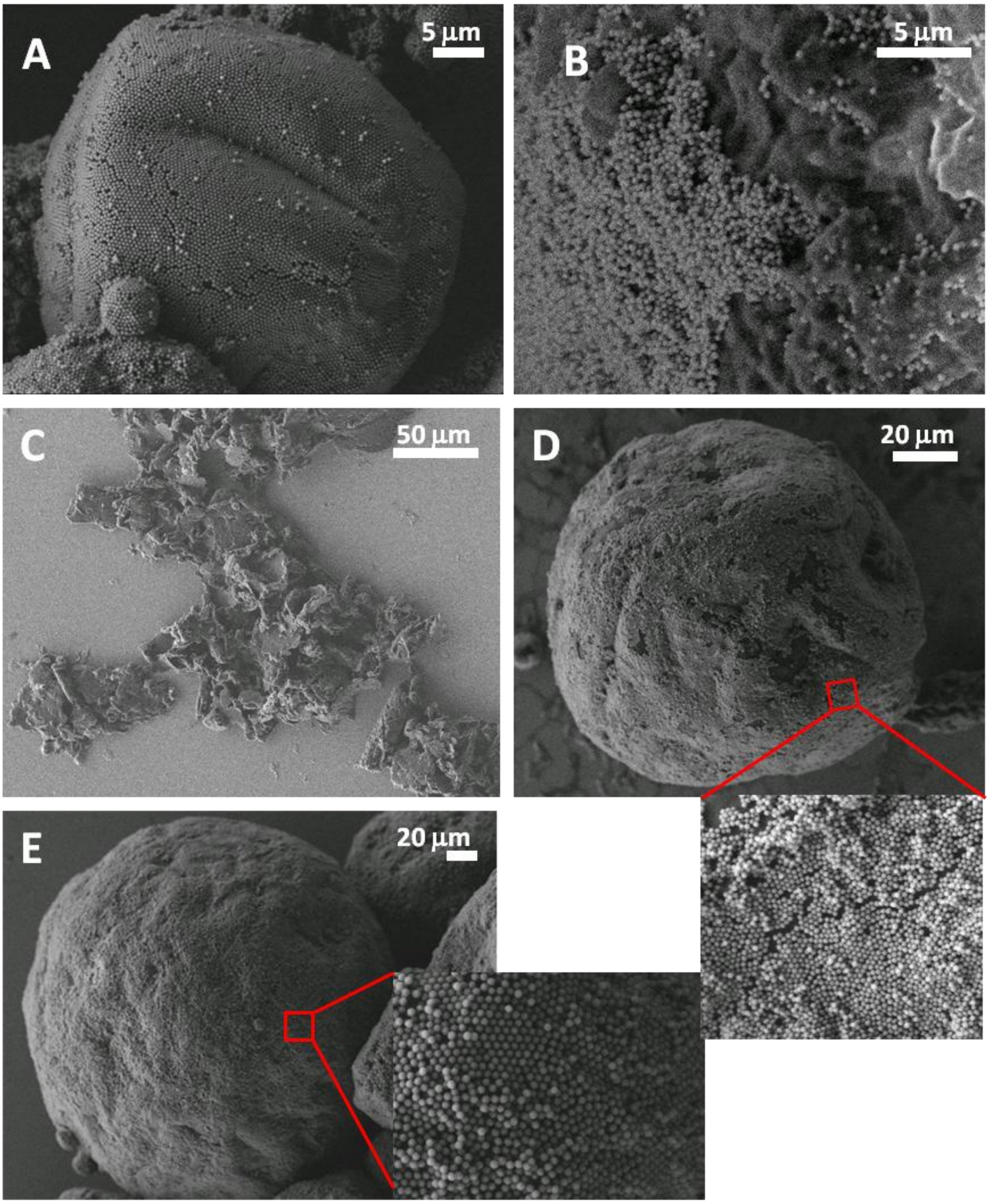

Figure S1. SEM micrographs of as prepared colloidosomes (**A**) and after: silanization processes (**B-E**). **(B and C)** Chemical modification in MeOH with 3-aminopropyltrimethoxysilane (APS) at room temperature for 20 min and 24 h., respectively. (**D**) equal to B but an ice bath. (**E**) Gas-phase silanization. In B and C colloidosomes are deformed or destroyed, and also present a lack of silica particles. In D there is only a slight deformation but the functionalization is not good. In E there is no deformation or lack of silica particles and functionalization is good enough for next steps.

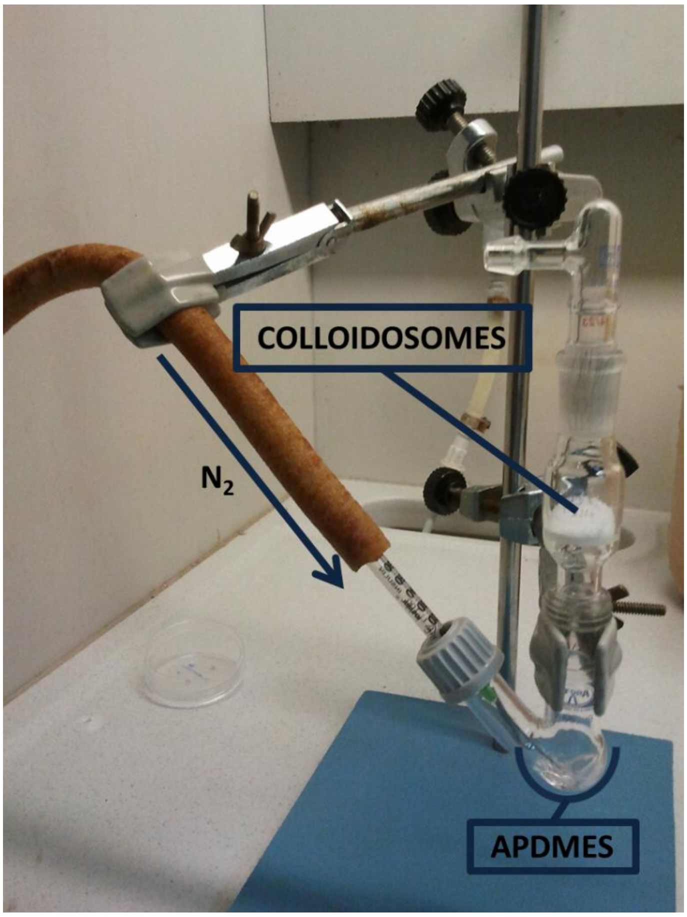

Figure S2. Experimental setup employed for the gas-phase silane deposition.

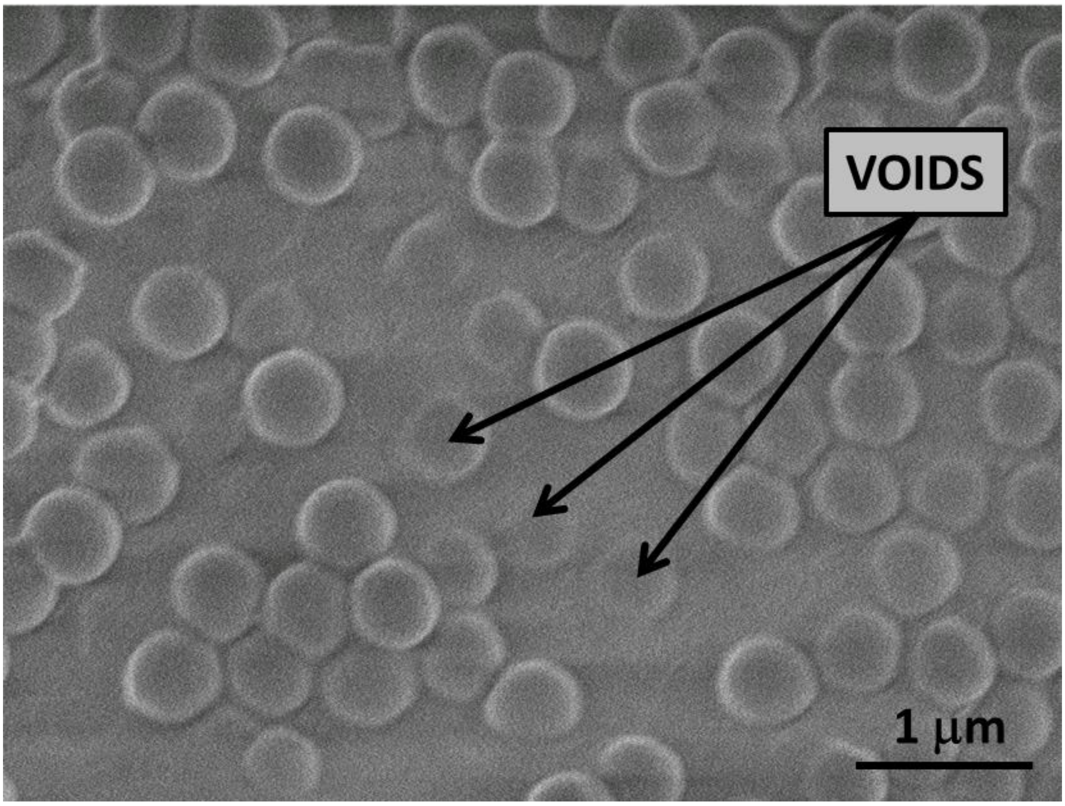

Figure S3. Cryo-SEM micrograph of 495 nm silica colloidosomes prepared using 60mg/L DDAB concentration. Voids left by the silica particles during the washing steps are indicated with arrows.

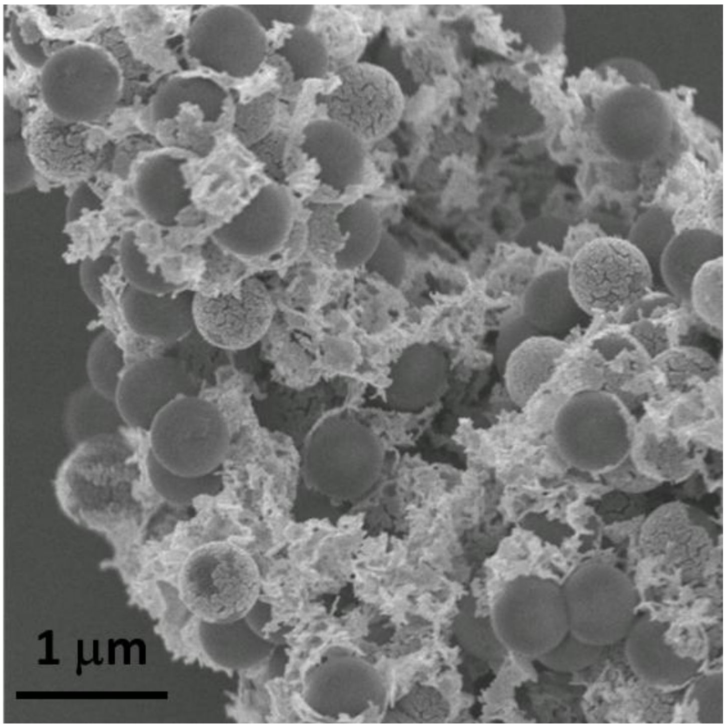

Figure S4. SEM micrograph of 495 non-homogeneous Au semishells obtained using a high ratio between Janus silica particles and Au^{3+} ions. Spikes are observed due to the fast reduction of gold ions.

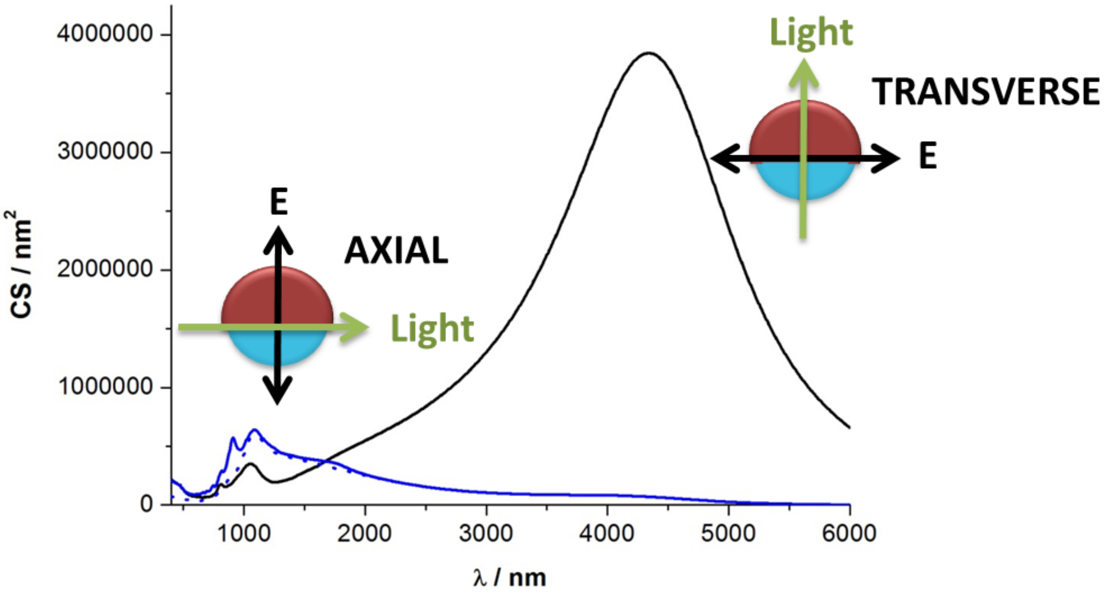

Figure S5. BEM simulations for 500 nm Au semishells with a gold layer of 10 nm. (**black)** Cross section spectrum calculated with the incident light polarized parallel to the axis of symmetry and therefore showing the transverse modes. (**blue)** Cross section spectrum calculated with the incident light polarized perpendicular to the axis of symmetry and therefore showing the axial modes. Full line was calculated with multipoles up to l=5, whereas dashed line correspond to l=1 (only dipoles).

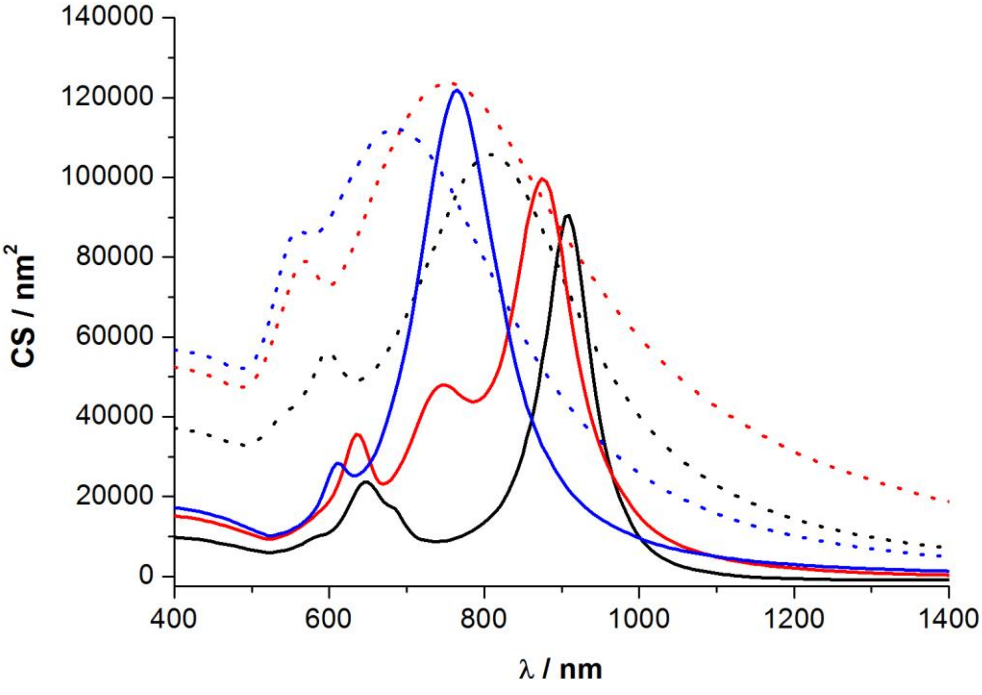

Figure S6. BEM simulations for Au semishells with 50% (black) and 75% (red) metal coverage, and nanoshells (complete shell, blue). The thickness of the gold shells was 10 nm (solid lines) and 30 nm (dotted lines). The diameter of the silica cores was 100 nm.
